# Effectiveness of analgesia with hydromorphone hydrochloride for postoperative pain following surgical repair of structural congenital malformations in children: a randomized controlled trial

**DOI:** 10.1186/s12871-021-01412-8

**Published:** 2021-07-16

**Authors:** Yongying Pan, Yimin Wang, Dongxu Lie, Di Liu, Xi Chen, Zeyan Wu, Liumei Chen, Huaizhen Wang, Liangming Peng, Huiying Liang, Xingrong Song, Baisong Zhao

**Affiliations:** 1 Department of Anesthesiology, Guangzhou Women and Children’s Medical Center, Guangzhou Medical University, No. 9 Jinsui Road, Tianhe District, Guangdong 510623 Guangzhou, China; 2 Department of Anesthesiology, Guangdong Second Provincial General Hospital, Guangzhou, 510000 China; 3 Institute of Pediatrics, Guangzhou Women and Children’s Medical Center, Guangzhou Medical University, No. 9 Jinsui Road, Tianhe District, Guangdong 510623 Guangzhou, China

**Keywords:** Analgesic effectiveness, Hydromorphone hydrochloride, Structural congenital malformation, Children, Randomized controlled trial

## Abstract

**Background:**

Effective postoperative analgesia is needed to prevent the negative effects of postoperative pain on patient outcomes. To compare the effectiveness of hydromorphone hydrochloride and sufentanil, combined with flurbiprofen axetil, for postoperative analgesia in pediatric patients.

**Methods:**

This prospective randomized controlled trial included 222 pediatric patients scheduled for repair of a structural congenital malformation under general anesthesia. Patients were randomized into 3 groups: hydromorphone hydrochloride 0.1 mg/kg (H1), hydromorphone hydrochloride 0.2 mg/kg; (H2) or sufentanil 1.5 µg/kg (S). Analgesics were diluted in 0.9% saline to 100 ml and infused continuously at a basic flow rate of 2 mL per h. The primary outcome measure was the Face, Legs, Activity, Cry, and Consolability (FLACC) pain score. Secondary outcomes included heart rate (HR), respiration rate (RR), SpO_2_, Ramsay sedation scores, scores on the Paediatric Anaesthesia Emergence Delirium (PAED) scale, adverse reactions, parent satisfaction with analgesia.

**Results:**

The FLACC score was significantly lower in H1 and H2 groups compared to S. The Ramsay sedation score was significantly higher in H1 and H2 groups compared to S. Recovery time was shorter in H1 group compared to patients H2 group or S group. There were no significant differences in the PAED scale, HR, RR, SpO2, adverse reactions, satisfaction of parents with analgesia, or length and cost of hospital stay.

**Conclusions:**

Hydromorphone hydrochloride is a more effective analgesic than sufentanil for postoperative pain in pediatric patients following surgical repair of a structural congenital malformation, however, hydromorphone hydrochloride and sufentanil had similar safety profiles in this patient population.

**Trial registration:**

Chinese Clinical Trial Register ChiCTR-INR-17013935). Clinical trial registry URL: Date of registration: December 14, 2017.

**Supplementary Information:**

The online version contains supplementary material available at 10.1186/s12871-021-01412-8.

## Background

Birth-defect refers to any structural or functional congenital anomaly, including cleft lip, cleft palate, hypospadias, and anus imperforate. Birth-defects are common, costly, and critical, affecting one in 33 births [1]. Early surgical repair is the primary treatment for structural congenital malformations. Surgical repair of structural congenital malformations can result in severe postoperative pain [2], which can cause sleep disturbances and changes in behavior, and negatively impact a child’s physical and mental health [3]. Several studies showed that regional anesthesia, maxillary nerve block, or pudendal or caudal block provided analgesia for repair of structural congenital malformations in children [4–6]. However, the results of these studies should be interpreted with caution. The analgesic effects of regional blocks could not be accurately assessed, as children underwent surgery under general anesthesia. The comparative effectiveness of analgesics cannot be evaluated, as the studies used different concentrations of local anesthetic [2–7].

Currently, intravenous analgesia is preferred for postoperative pain control following repair of structural congenital malformations. Opioids are effective as analgesics and have widespread medical use. Sufentanil, a selective μ-receptor agonist is often administered for pediatric postoperative analgesia because of its rapid peak, potent analgesic effect and short half-life [8]. Hydromorphone, a semisynthetic μ and δ opioid agonist, is also commonly used as it is effective in achieving analgesia, has no ceiling effect for analgesia, and has less side effects than morphine [9]. Studies have shown that treatment of postoperative pain is inadequate in pediatrics, mainly due to concern over opioid-related adverse effects [10]. Multimodal analgesic regimens may provide satisfactory postoperative analgesia and minimize those adverse effects.

Studies investigating the effectiveness of sufentanil or hydromorphone for analgesia in pediatric patients undergoing surgery for repair of structural congenital malformations are scarce. This randomized controlled trial compared the effectiveness of hydromorphone hydrochloride and sufentanil, combined with flurbiprofen axetil, for postoperative analgesia in pediatric patients undergoing surgical repair of structural congenital malformations [11, 12]. Two doses of hydromorphone hydrochloride were used to determine which dose of hydromorphone hydrochloride was most effective for postoperative pain but associated with the fewest adverse events. Flurbiprofen axetil is an injectable nonsteroidal anti-inflammatory drug (NSAID) that provides an excellent analgesic effect in multimodal analgesia after various pediatric procedures [13, 14].

## Methods

### Study design

This prospective randomized clinical trial was approved by the Ethics Committee of Guangzhou Women and Children’s Medical Center (protocol No. 2017091701), and written informed consent was obtained from parents or legal guardians of the children included in the trial.

This study adheres to CONSORT guidelines and the authors have completed the CONSORT checklist.

### Participants

Children undergoing surgical repair of a structural congenital malformation under general anesthesia at Guangzhou Women and Children’s Medical Center between February 2018 and June 2018 were eligible for this trial. Inclusion criteria were 1) American Society of Anesthesiologists (ASA) physical status I or II and 2) aged 6 months to 3 years. Exclusion criteria were 1) premature infants;‘ 2) severe obesity; 3) severe sleep apnea syndrome; 4) history of arrhythmia, bronchial asthma, acute upper respiratory infection, or presence of or potential for difficult airways; 5) history of hepatitis or renal dysfunction; 6) abnormal recovery from anesthesia after a previous surgery; 7) history of chronic pain and long-term use of analgesics; 8) use of analgesic, sedative or antipruritic drugs or quinolone antibiotics 24 h before the operation; 9) history of an allergic reaction to hydromorphone hydrochloride, flurbiprofen axetil, opioids or their components; or 10) history of mental and neurological disease, which may have caused issues when evaluating the effectiveness of analgesia.

### Randomization and blinding

Patients were randomized 1:1:1 using a computer-generated randomization table into 3 groups: hydromorphone hydrochloride 0.1 mg/kg (H1, *n* = 74), hydromorphone hydrochloride 0.2 mg/kg (H2, *n* = 74), or sufentanil 1.5 µg/kg (S, *n* = 74). All patients received flurbiprofen axetil 5 mg/kg. Analgesics were diluted in 0.9% saline to 100 ml and infused continuously at a basic flow rate of 2 mL per h. The researchers and other medical staff in the operation room were blinded to the group allocation until after the study had been completed. The child, their parents or legal guardians and family, and the nurses on the ward were not blinded to the group allocation.

### Perioperative Management

Preoperatively, propofol 1.0 mg/kg was injected intravenously for sedation. Routine monitoring included electrocardiogram (ECG), heart rate (HR), noninvasive blood pressure (BP), pulse oximetry, and end-tidal CO_2_. After peripheral intravenous access was established, and prior to incision, patients received an infusion of cisatracurium (0.2 mg/kg), sufentanil 0.3 ug/kg, propofol (2.5–3.0 mg/kg), and flurbiprofen axetil (1 mg/kg). Three minutes later, patients were provided endotracheal or laryngeal mask airway intubation. Mechanical ventilation was administered. Anesthesia was maintained with 2%-3% sevoflurane. Boluses of sufentanil and a continuous intravenous infusion of propofol were used to maintain an adequate depth of anesthesia. Boluses of cisatracurium were used to ensure muscle-relaxation. Vasoactive drugs were administered to maintain intraoperative BP and HR within 20% of baseline. No nerve block or local infiltration was used for cleft lip or palate surgery. Sufentanil and cisartacurium were discontinued 30 min before the end of surgery. Tropisetron 0.1 mg/kg was administered by intravenous injection. Administration of analgesia via an intravenous pump was initiated 10 min before the end of surgery, and continued for 48 h after surgery. Sevoflurane and other continuous infusions were discontinued at the end of surgery. Patients were transferred to the post-anesthesia care unit (PACU). The tracheal tube or laryngeal mask airway was extubated after recovery of consciousness and spontaneous breathing. Supplemental oxygen was provided, and patients were monitored for 30 min in the PACU.

### Outcome measures

The primary outcome measure was the Face, Legs, Activity, Cry, and Consolability (FLACC) pain score (scales of 0–2) at the time of leaving the PACU and 2 h, 6 h, 12 h, 24 h, 36 h, and 48 h after surgery [15]. Secondary outcomes included HR, respiration rate (RR), SpO_2_ and Ramsay sedation scores, where a score of 2–4 was regarded satisfactory sedation [16] at the time of leaving the PACU and 2 h, 6 h, 12 h, 24 h, 36 h, and 48 h after surgery. Scores on the Paediatric Anaesthesia Emergence Delirium (PAED) scale (5 psychometric items scored on a scale from 0–4) [17], the FLACC pain score, and the Ramsay sedation score were recorded after extubation. Adverse reactions (postoperative nausea and vomiting [PONV], pruritus), parent satisfaction with analgesia (1, highly satisfied; 2, satisfied; 3, unsatisfied), and the length and cost of the hospital stay were also recorded.

### Statistical analysis

Based on the expected effective sample size and preliminary test results, we determined that 50 patients per group were needed to achieve a power of 0.8 with a significance level (2-sided) of *P* = 0.05 (www.stats.ox.ac.uk/~snijders/multilevel.htm).

Statistical analyses were performed using R statistical software (v3.6.1, R Language). For time-invariant continuous variables, the Shapiro test was used to test the normality assumption, and Levene’s test was used to test the homoscedasticity assumption. Data that retained both assumptions were analyzed by the F-test to compare group-wise means. Otherwise, the Kruskall-Wallis test with Hodges Lehman estimator was used to compare group-wise medians. Time invariant categorical data were analyzed with the Chi-square test. A mixed effect model was used to evaluate the FLACC pain score, Ramsay sedation score, HR, RR, and SpO2. An approximate F-test based on the Kenward-Roger approach was used to determine group-wise differences. For data after extubation, the Kruskal–Wallis test was used to compare differences in recovery time, length and cost of hospital stay, emergence delirium, and the emergence agitation total score. Satisfaction with analgesia and adverse reactions were compared with the Chi-square test. Statistical significance was set at *P* < 0.05.

## Results

This study enrolled 228 patients. Six patients did not receive the intervention; therefore, data from 222 patients were included in the analyses (n = 74 in each group) (Supplementary Fig. 1). There were no significant differences in demographic characteristics or type of surgery between the three groups. There were no significant differences in duration of analgesia or surgery, amount of propofol, sevoflurane, sufentanil, cisatracurium, tropisetron, penehyclidine hydrochloride or flurbiprofen axetil administered in the intraoperative period, or length and cost of hospital stay between the three groups (Table 1).

**Table 1:** Demographic and perioperative characteristics

**Variable**	**Group**			
	**S** **N = 74**	**H1** **N = 74**	**H2** **N = 74**	***P* value (*K-W Test*)**
**Age (month)**; mean ± SD	14.93 ± 9.39	18.95 ± 12.79	15.19 ± 8.70	0.277
**Gender (male)**	50(67.57%)	46(62.16%)	51 (68.92%)	0.655
**Weight (kg)**; mean ± SD	9.35 ± 2.15	10.33 ± 3.03	9.97 ± 2.38	0.096
**ASA (II)**^a^	74(100%)	74(100%)	74(100%)	1
**Surgery**				0.980 (Chi Square Test)
Urinary system malformation repair	25(33.78%)	25(33.78%)	26(35.14%)	
Cleft lip, cleft palate or both repair	49(66.22%)	49(66.22%)	48(64.86%)	
**Administration of drug during the operation**				
Propofol (mg); mean ± SD	30.54 ± 8.82	32.84 ± 7.77	30.86 ± 9.80	0.059
Sulfentanil (mcg); mean ± SD	3.47 ± 0.94	3.73 ± 0.99	3.50 ± 1.20	0.116
Cisatracurium (mg); mean ± SD	2.64 ± 0.72	2.51 ± 0.97	2.54 ± 1.06	0.733
Tropisetron (mg); mean ± SD	1.11 ± 0.86	1.07 ± 0.28	1.00 ± 0.200	0.272
Penehyclidine hydrochloride (mg); mean ± SD	0.05 ± 0.09	0.04 ± 0.05	0.04 ± 0.05	0.926
Flurbiprofen axetil (mg); mean ± SD	10.01 ± 2.06	10.51 ± 2.89	10.00 ± 1.76	0.239
** Duration of surgery (min)**; mean ± SD	78.23 ± 45.79	67.07 ± 33.20	85.58 ± 55.09	0.126
** Duration of anesthesia (min)**; mean ± SD	107.70 ± 50.19	100.22 ± 41.05	104.84 ± 59.48	0.694
** Recovery time (min)**; mean ± SD	17.70 ± 5.82	16.28 ± 5.25	18.14 ± 5.62	0.033
** Length of hospital (day)**; mean ± SD	5.39 ± 3.33	5.14 ± 2.10	5.39 ± 3.27	0.812
** Cost of hospital (thousand Yuan)**; mean ± SD	16.76 ± 7.78	14.79 ± 4.03	16.38 ± 6.87	0.680

^a^*ASA* American Society of Anesthesiologists. Patients were randomized 1:1:1 into 3 groups: hydromorphone hydrochloride 0.1 mg/kg (H1), hydromorphone hydrochloride 0.2 mg/kg (H2), or sufentanil 1.5 µg/kg (S). All patients received flurbiprofen axetil 5 mg/kg

Results from the mixed effect model showing the differences in the FLACC pain score, Ramsay sedation score, HR, RR, and SpO2 between the three groups are shown in Supplementary Table 1.

**Fig. 1:**
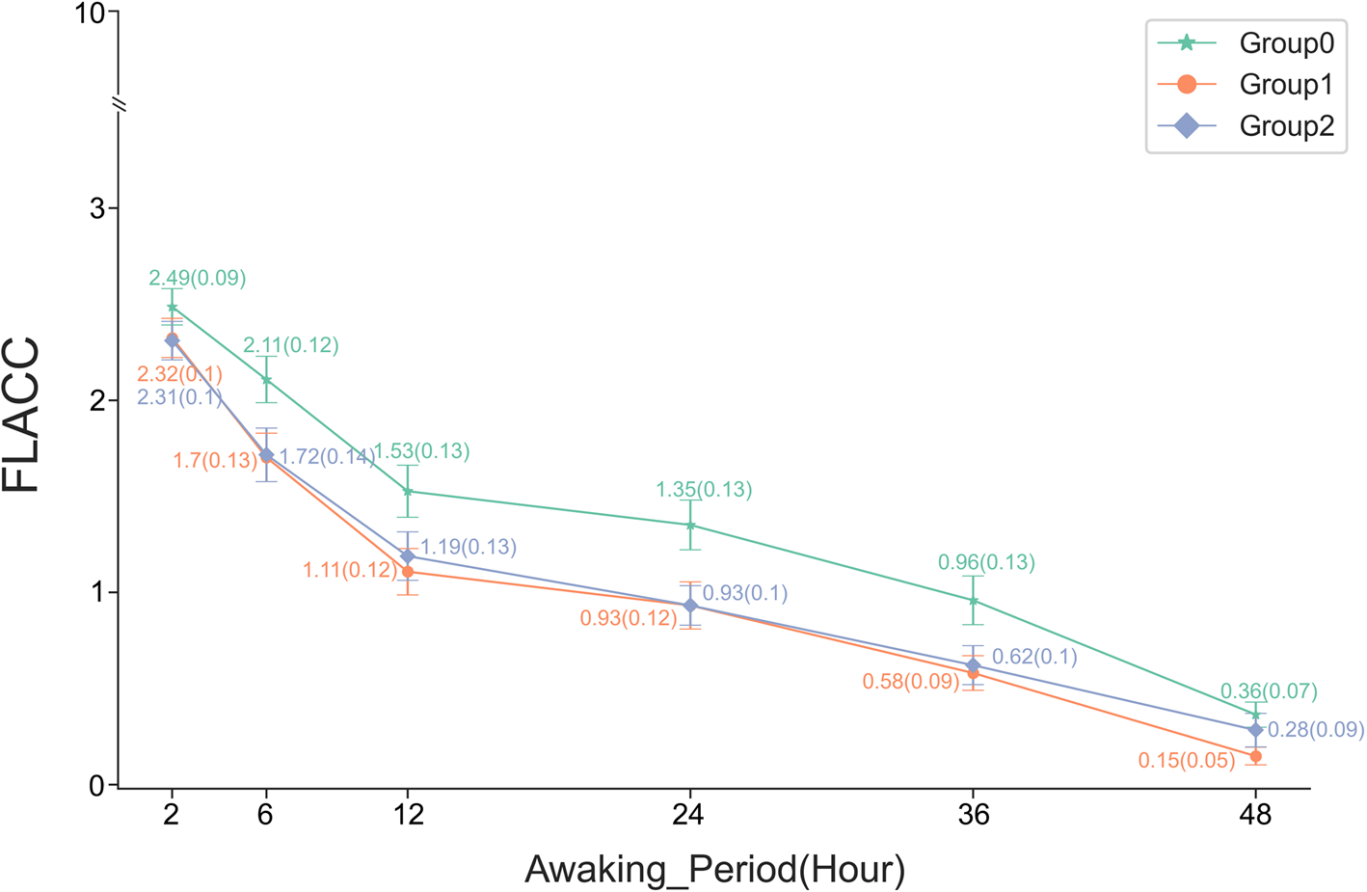
Postoperative FLACC scores

The FLACC pain score was significantly lower in patients who received hydromorphone hydrochloride 0.1 mg/kg (*P* < 0.01) or hydromorphone hydrochloride 0.2 mg/kg (*P* = 0.01) compared to patients who received sufentanil 1.5 µg/kg. There was no significant difference in FLACC pain score in patients who received hydromorphone hydrochloride 0.1 mg/kg or hydromorphone hydrochloride 0.2 mg/kg (Fig. 1).

**Fig. 2:**
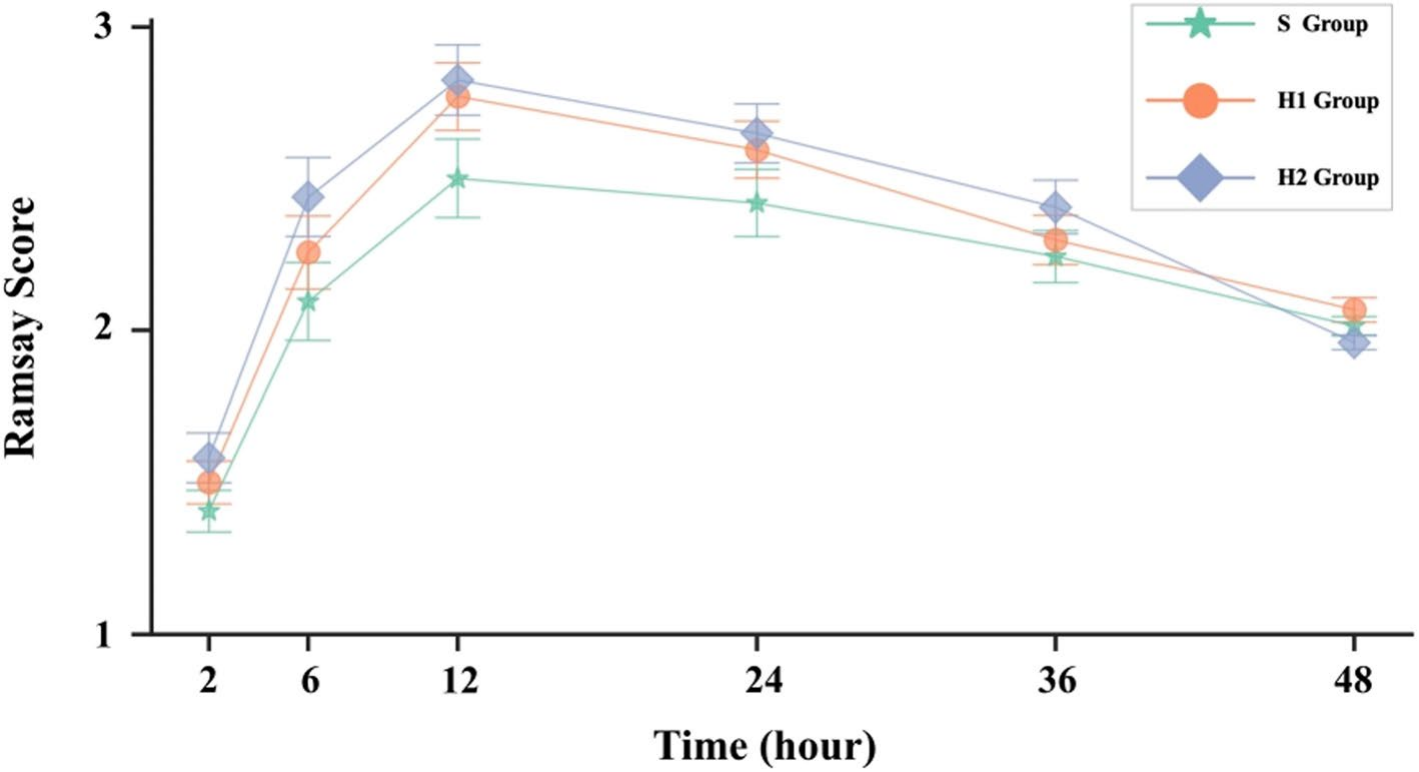
Postoperative Ramsay sedation scores

The Ramsay sedation score was significantly higher in patients who received hydromorphone hydrochloride 0.2 mg/kg compared to patients who received sufentanil 1.5 µg/kg (*P* < 0.01). There was no significant difference in the Ramsay sedation score in patients who received hydromorphone hydrochloride 0.2 mg/kg or sufentanil 1.5 µg/kg compared to patients who received hydromorphone hydrochloride 0.1 mg/kg (Fig. 2).

Recovery time was shorter in patients who received hydromorphone hydrochloride 0.1 mg/kg compared to patients who received hydromorphone hydrochloride 0.2 mg/kg (16.28 ± 5.25 vs. 18.14 ± 5.62 min) or sufentanil 1.5 µg/kg (16.28 ± 5.25 vs. 17.70 ± 5.82 min) (Table 1).

**Fig. 3:**
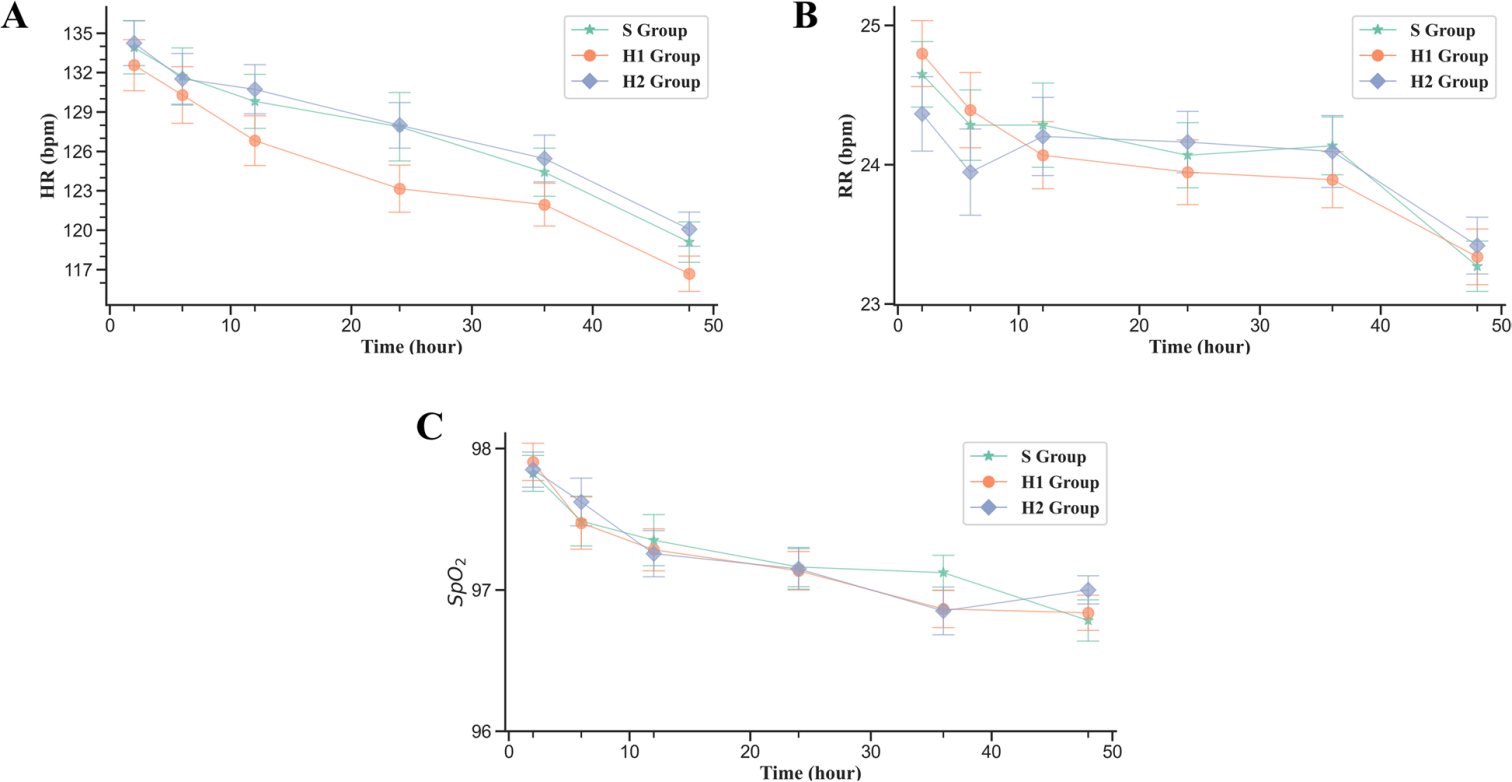
Postoperative HR, RR, and SpO_2_

There were no significant differences in the PAED scale, the FLACC pain score, or the Ramsay sedation score after extubation (Table 2). There were no significant differences in HR, RR, SpO2 (Fig. 3), adverse reactions, or satisfaction of parents with analgesia (Table 3).

**Table 2:** FLACC, Ramsay and PAED scores in the post anesthesia care unit

Variable	Group			*P* value (Chi Square Test)
	**S** **N = 74 (%)**	**H1** **N = 74 (%)**	**H2** **N = 74 (%)**	
**PAED total score**	4.69 ± 4.17	4.20 ± 3.89	4.96 ± 3.86	0.470(K-W Test)
**FLACC score**				0.849
0	0(0)	2(2.70)	2(2.70)	0.361
1	8(10.81)	15(20.27)	3(4.05)	0.009
2	34(45.95)	22(29.73)	34(45.95)	0.069
3	31(41.89)	32(43.24)	35(47.30)	0.789
4	1(1.35)	2(2.70)	0(0)	0.363
5	0(0)	1(1.35)	0(0)	0.366
**Ramsay score**				0.278
1	34(45.95)	32(43.24)	30(40.54)	0.802
2	36(48.65)	33(44.59)	30(40.54)	0.611
3	4(5.41)	9(12.16)	13(17.57)	0.070
4	0(0)	0(0)	1(1.35)	0.366

Patients were randomized 1:1:1 into 3 groups: hydromorphone hydrochloride 0.1 mg/kg (H1), hydromorphone hydrochloride 0.2 mg/kg (H2), or sufentanil 1.5 µg/kg (S). All patients received flurbiprofen axetil 5 mg/kg

**Table 3:** Comparison of postoperative complications

	**Group**			***Statistics***	***P* value** **(**Chi Square Test**)**
	**S** **N = 74 (%)**	**H1** **N = 74 (%)**	**H2** **N = 74 (%)**		
Complications				0.497	0.974
None	43(58.10)	43(58.11)	42(56.75)	0	1.000
PONV	27(36.49)	28(36.84)	27(36.49)	0.038	0.981
Pruritus	4(5.41)	3(3.95)	5(6.76)	0.529	0.768
Satisfaction				8.373	0.079
1	11(14.86)	10(13.54)	21(28.3)	6.519	0.038
2	58(78.38)	62(83.78)	51(68.92)	4.735	0.094
3	5(6.76)	2(2.70)	2(2.7)	2.085	0.353

Patients were randomized 1:1:1 into 3 groups: hydromorphone hydrochloride 0.1 mg/kg (H1), hydromorphone hydrochloride 0.2 mg/kg (H2), or sufentanil 1.5 µg/kg (S). All patients received flurbiprofen axetil 5 mg/kg

## Discussion

To the author’s knowledge, this is the first randomized, single-blind controlled trial comparing hydromorphone hydrochloride with sufentanil, in combination with flurbiprofen axetil, for postoperative analgesia following surgical repair of structural congenital malformations in a pediatric patient population. Findings showed that pediatric patients administered hydromorphone hydrochloride had a lower FLACC pain score, especially 6 to 24 h after surgery, better pain relief, and a shorter recovery time compared to those administered sufentanil (1.5 µg/kg).

Currently, hydromorphone and sufentanil are used widely in clinical anesthesia and postoperative analgesia. Most studies have reported on the analgesic effects of hydromorphone and sufentanil and the incidence of adverse reactions in adults. Yanqing Yang et al. showed hydromorphone and sufentanil had similar analgesic effects in adults undergoing elective laparoscopic or open radical surgery[18].

In the present study, pediatric patients received multimodal analgesia consisting of hydromorphone hydrochloride or sufentanil in combination with flurbiprofen axetil to improve patient safety and quality of surgical care [19]. Flurbiprofen axetil is an NSAID with high affinity to the site of surgical incision and inflammatory tissues, which exerts its effects through peripheral and central mechanisms and has synergistic analgesic effects with opiods [19, 20]. In a previous study, flurbiprofen axetil enhanced the analgesic effects of sufentanil, decreased postoperative opioid consumption, and attenuated emergence agitation and systemic proinflammation in adults undergoing tangential excision surgery [21].

Evidence suggests that parental hydromorphone hydrochloride is 5–6.7 times as potent as morphine sulfate [22], parenteral sufentanil is 1000 times as potent as morphine sulfate, and sufentanil is 150–200 times as potent as hydromorphone hydrochloride. Therefore, in the present study, we estimate that sufentanil 1.5 µg/kg was 150 times as potent as hydromorphone hydrochloride 0.1 mg/kg and 75 times as potent as hydrochloride 0.2 mg/kg. However, our results showed that hydromorphone hydrochloride was a more effective analgesic than sufentanil. This may be because hydromorphone has a high affinity for μ and δ-opioid receptors, which possess strong analgesic activities[23]. Of note, hydromorphone administered by target-controlled infusion and patient-controlled analgesia (PCA) to postoperative adult cardiac surgery patients improved mood, which is closely related to pain relief, likely due to the anti-anxiety and the anti-depression effects induced by activation of δ-opioid receptors. Sufentanil’s short duration of analgesia may be explained by its low affinity for the δ-opioid receptor and rapid redistribution [24].

In the present study, the Ramsay sedation score was significantly higher in patients who received hydromorphone hydrochloride 0.2 mg/kg compared to patients who received sufentanil 1.5 µg/kg. This was likely mediated by the action of hydrophone on the δ-opioid receptor in the central nervous system, which produces a sedative effect. The FLACC score, which considers five categories including Face, Legs, Activity, Cry, and Consolability, is an objective measure of pain that will have minimized the effect of sedation level on our findings.

Common side effects associated with opioids include respiratory depression, PONV, pruritus, and excessive sedation. In our patient population, there were no significant differences in the incidence of PONV or pruritus or in postoperative HR, RR or SpO_2_ in patients who received hydromorphone hydrochloride 0.1 mg/kg, hydromorphone hydrochloride 0.2 mg/kg, or sufentanil 1.5 µg/kg, and no patients experienced respiratory depression. PONV is influenced by various factors, including the anesthetic, surgical approach, and patient demographic and clinical characteristics [25]. In the present study, were no significant differences in age, gender, body weight, type of surgery, operative or anesthesia time, or intraoperative opioid or other drug use in patients who received hydromorphone hydrochloride 0.1 mg/kg, hydromorphone hydrochloride 0.2 mg/kg, or sufentanil 1.5 µg/kg. These data confirm the safety of hydromorphone hydrochloride or sufentanil for postoperative analgesia in pediatric patients undergoing surgical repair of structural congenital malformations. Consistent with this, in a previous study, pediatric patients who received a PCA were more likely to switch from morphine-to-hydromorphone than vice-versa as hydromorphone provided improved pain control and fewer side effects [9].

This study was associated with several limitations. First, we only included children with structural congenital malformations of the oral cavity or urinary tract; other congenital malformations will be considered in future studies. Second, some children were administered an intravenous injection of propofol as they were distressed at the prospect of surgery. Therefore, it was difficult for anesthesiologists to accurately record these patients’ preoperative HR, RR, and SPO_2_. Third, as children are unable to safely activate PCA independently, the use of rescue analgesia was controlled by the researchers or parents. No patients required rescue analgesia in this study. Last, the study was not powered to look at the side-effects of the pain control methods.

## Conclusions

In conclusion, hydromorphone hydrochloride is a more effective analgesic than sufentanil for postoperative pain in pediatric patients following surgical repair of a structural congenital malformation under general anesthesia; however, hydromorphone hydrochloride and sufentanil had similar safety profiles in this patient population (side-effects of the pain control methods were not evaluated). We recommend hydromorphone hydrochloride 0.1 mg/kg for postoperative pain in pediatric patients following surgical repair of a structural congenital malformation, as this dose was associated with better recovery and more consistent sedation than hydromorphone hydrochloride 0.2 mg/kg.

## Supplementary Information

Additional file 1: **Figure 1.** CONSORT diagram.

Additional file 2: **Table 1.** F-statistic for RR, HR and SpO_2_, andFLACC and Ramsay.

## Data Availability

The datasets used and/or analysed during the current study are available from the corresponding author on reasonable request.

## Abbreviations

FLACC: Face, legs, activity, cry, and consolability pain score
HR: Heart rate
RR: Respiration rate
PAED: Paediatric Anaesthesia Emergence Delirium
NSAID: Nonsteroidal anti-inflammatory drug
ASA: American Society of Anesthesiologists
ECG: Electrocardiogram
BP: Blood pressure
PACU: The post-anesthesia care unit
PONV: Postoperative nausea and vomiting
PCA: Patient-controlled analgesia
