# Supplementary figures and images for: Effectiveness of analgesia with hydromorphone hydrochloride for postoperative pain following surgical repair of structural congenital malformations in children: a randomized controlled trial
Source: BMC Anesthesiol. 2021 Jul 16;21:192. doi: 10.1186/s12871-021-01412-8 (PMC8284015; doi:10.1186/s12871-021-01412-8)

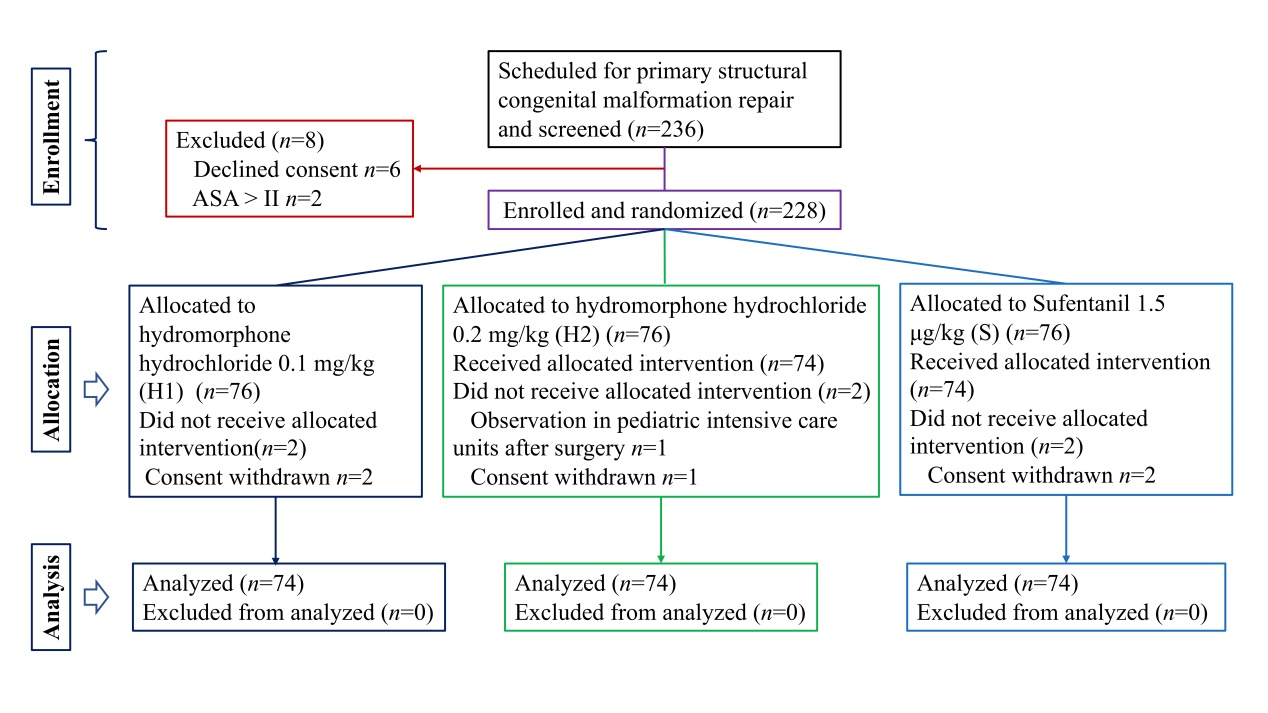

Supplement: Supplementary file 1 — Figure 1. CONSORT diagram. [file 12871_2021_1412_MOESM1_ESM.tif]
