# Supplementary material for: Effectiveness of analgesia with hydromorphone hydrochloride for postoperative pain following surgical repair of structural congenital malformations in children: a randomized controlled trial
Source: BMC Anesthesiol. 2021 Jul 16;21:192. doi: 10.1186/s12871-021-01412-8 (PMC8284015; doi:10.1186/s12871-021-01412-8)
Supplement: Supplementary file 2 — Table 1. F-statistic for RR, HR and SpO2, andFLACC and Ramsay. [file 12871_2021_1412_MOESM2_ESM.doc]

**Supplementary Table 1 F-statistic for RR, HR and SpO_2_, and FLACC and Ramsay scores**

| **Variable** | ***F* Statistics** | ***P* Value** |
| --- | --- | --- |
| RR | 0.101 | 0.75 |
| HR | 0.053 | 0.82 |
| SpO2 | 0 | 1 |
| FLACC | 6.769 | 0.01 |
| S group | 8.65 | <0.01 |
| H1 group | 6.51 | 0.01 |
| H2 group | 0.165 | 0.686 |
| Ramsay | 8.78 | <0.01 |
| S group | 4.178 | 0.04 |
| H1 group | 8.07 | <0.01 |
| H2 group | 0.892 | 0.347 |
